# Normal opening pressure lumbar puncture in cryptococcal meningitis complicated by a cerebral venous thrombosis: a case report and literature review

**DOI:** 10.3389/fmed.2025.1619155

**Published:** 2025-10-14

**Authors:** Vincent Tee, Abdul Hakim Maiden, Fatimah Othman, Ping Sheng Chua, Mung Seong Wong

**Affiliations:** ^1^Medical Department, Hospital Segamat, Segamat, Johor, Malaysia; ^2^Faculty of Medicine and Health Sciences, Universiti Putra Malaysia, Serdang, Malaysia

**Keywords:** opening pressure, cryptococcal, meningitis, venous thrombosis, normal pressure

## Abstract

Cryptococcal meningitis (CM) is a severe fungal infection that primarily affects individuals with compromised immune systems, such as those living with HIV/AIDS or undergoing immunosuppressive therapy. Elevated intracranial pressure is a common complication of CM and remains an important diagnostic parameter in patients with CM. This report describes a case of CM complicated with cerebral venous thrombosis (CVT) in an immunocompetent patient residing in the southern region of Peninsular Malaysia. The diagnosis of CM was confirmed by cerebrospinal fluid (CSF) culture; however, the patient presented with normal opening pressure during lumbar puncture. The pathophysiology and factors of causality, together with their diagnostic challenges, are discussed.

## 1 Introduction

Cryptococcal meningitis (CM) is a severe fungal infection that primarily affects individuals with compromised immune systems, such as those living with HIV/AIDS or undergoing immunosuppressive therapy. Globally, approximately 1 million cases of CM are reported annually, the majority of which are associated with AIDS. Approximately 6% of patients with AIDS develop cryptococcosis, and 85% of all cryptococcosis cases are associated with AIDS (1, 2). Elevated intracranial pressure (ICP) is one of the most common complications of CM and has been reported in >50% of patients (3).

CM is caused by the encapsulated yeast *Cryptococcus neoformans*, which is commonly found in the environment, particularly in soil contaminated with bird droppings. In immunocompromised individuals, such as those with low CD4 + T-cell counts, the fungus can disseminate from the lungs to the central nervous system, resulting in meningitis (2, 4). However, isolated cases in immunocompetent patients suggest alternative mechanisms of infection or a subtle immune dysfunction predisposing them to the disease.

The clinical presentation of CM can vary, making diagnosis challenging, especially in immunocompetent patients. Common symptoms, often due to high intracranial pressure, include headache, fever, neck stiffness, vomiting, blurring of vision, photophobia, and altered mental status. However, the disease may progress insidiously with atypical presentations such as isolated cranial nerve palsies or focal neurological deficits. A high index of suspicion is crucial for early diagnosis and prompt initiation of appropriate treatment.

The diagnosis of CM relies on the detection of *Cryptococcus neoformans* in cerebrospinal fluid (CSF) through microscopy, culture, or antigen detection assays. Lumbar puncture is mandatory for diagnosing CM and often reveals high opening pressure due to increased intracranial pressure. The pathophysiology of raised ICP in CM is believed to be caused by obstruction of the reabsorption of CSF at the arachnoid villi by *Cryptococcus*, which can lead to communicating hydrocephalus (4). Therefore, magnetic resonance imaging (MRI) can aid in assessing the extent of CNS involvement and identifying complications such as hydrocephalus or intracranial mass effect.

The management of CM involves a combination of antifungal therapy and supportive care. Induction therapy typically consists of amphotericin B-based regimens, followed by consolidation and maintenance therapy with oral fluconazole. Adjunctive therapies, including corticosteroids, may be considered in certain patients to reduce inflammation and improve outcomes. Repetitive lumbar puncture is one of the management strategies in CM to reduce the opening pressure lumbar puncture below 20 mmHg, thereby alleviating patient symptoms.

This case report describes the clinical course, diagnostic challenges, and therapeutic approach in an immunocompetent patient with normal opening pressure lumbar puncture cryptococcal meningitis. By highlighting this unusual presentation, we aim to emphasize the need for clinicians to maintain a broad differential diagnosis, even in patients without apparent immunosuppression and normal opening lumbar pressure, to facilitate early recognition and appropriate management of this potentially life-threatening infection.

## 2 Case description

We present the case of a 34-year-old Malay man residing in the Segamat district of Johor, Malaysia. He has no known medical history and maintains a healthy body weight, estimated at 75 kg. The patient has worked as a farmer for a few years. On 3 October 2023, he presented at our center with a 3-day history of fever and a sudden onset of right-sided headache occurring in on-and-off episodes daily. The headache, aggravated by position or diurnal variation, was described as throbbing with a pain score of 7 out of 10, lasted for 3 h, and was slightly relieved by paracetamol. He also reported associated symptoms of blurred vision, photophobia, and neck pain, which had been ongoing for 1 week. There were no occurrences of body weakness, seizures, aura, upper respiratory tract infection (URTI) symptoms, abdominal pain, or loose stools. There were no recent history of trauma or fall. He was a non-smoker and did not consume alcohol. He denied taking traditional medication or over-the-counter medicines and reported no high-risk behaviors. He had no previous history of hospitalization and had been well for 1 week prior to the admission. Moreover, there was no family history of malignancy or similar illnesses.

Upon arrival at the emergency department, he was alert, his blood pressure (BP) was 150/91 mmHg, heart rate (HR) was 95 bpm, oxygen saturation (SpO2) was 100% on room air (RA), and his temperature was 36.5 °C. On examination, the findings were normal for the cardiovascular and respiratory systems. A full neurological examination revealed normal findings, and the ocular assessment by the ophthalmology team was also normal. Hemoglobin was 17.2, total white cell count was 10.8, platelet count was 192, hematocrit was 52.8, and C-reactive protein was 9.58. The renal profile and liver function tests were normal. Viral screening was negative for HIV, Hepatitis B, and Hepatitis C. Antinuclear antibody was negative, and C3 and C4 levels were normal. The antiphospholipid antibody was negative, and the blood culture and sensitivity showed no growth. A contrast-enhanced computed tomography (CECT) scan of the brain revealed superior sagittal sinus thrombosis. He was initially treated as a case of meningoencephalitis and started on IV Rocephin 2 g BD and IV Acyclovir 500 mg TDS, and S/C Clexane 60 mg BD. Due to clinical indications pointing toward meningitis, a lumbar puncture was performed on 4 October 2023. Although the opening pressure was only 15 mmHg, the routine CSF investigation yielded unexpected results, as shown in Table 1.

**Table 1:** CSF investigations.

CSF investigations	Results
CSF biochemistry	Glucose 0.6 (CSF glucose to serum glucose ratio 0.1), protein 1.1 (CSF protein to serum protein ratio 0.015)
CSF full and microscopic examination	Clear and colorless, cell count 9 WBC/μL, polymorph 0, Gram stain: no organism seen, Indian ink negative.
CSF culture	*Cryptococcus neoformans*
CSF mycobacterium tuberculosis C + S	No Growth
CSF cryptococcal antigen	Detected (titer 1:32768)
CSF polymerase chain reaction meningitis/encephalitis viral panel	*Cryptococcus neoformans* detected
CSF cytology	Smears show a rounded budding fungal yeast with a thick refractile capsule. The background consists of lymphocytes, monocytes, and red blood cells. Suggestive of cryptococcal meningitis

Treatment for cryptococcal meningitis was started on 6 October 2023 (turnaround time of 3 days) after the CSF cryptococcal PCR test was noted to be positive. IV Amphotericin B and oral Flucytosine were started immediately. Subsequently, a repeat lumbar puncture was performed on day 2 of antifungal treatment due to persistent headache and photophobia. During this procedure, a different operator was involved, and the opening pressure remained low at 8 mmHg.

He was discharged in stable condition after completing a two-week induction therapy with IV amphotericin B. Upon discharge, he was prescribed oral tablets of fluconazole 800 mg once daily (OD) for 8 weeks, followed by oral tablets of fluconazole 200 mg OD for 1 year. Subsequent follow-ups at our outpatient clinic showed no recurrence of symptoms.

## 3 Discussion

This case report describes a 34-year-old immunocompetent man with CM who had a normal lumbar puncture opening pressure of 15 mmHg. This is considered an unusual finding, as more than 50% of CM cases are associated with elevated intracranial pressure (ICP). A prompt lumbar puncture to measure baseline opening pressure is generally recommended for CM. However, if focal neurological deficits or altered mental status are present, the procedure should be delayed until appropriate radiological imaging is performed. Treatment typically involves therapeutic CSF pressure reduction via lumbar puncture, which may be repeated if symptoms persist or until the pressure remains stable for over 2 days. In cases requiring frequent lumbar punctures, alternative interventions such as temporary percutaneous lumbar drains or ventriculostomy may be necessary (5).

The diagnostic challenges faced in this case may have led clinicians to false reassurance, typically in a tropical country like Malaysia, as a normal opening pressure delayed CM suspicion, leading to an initial treatment for bacterial or viral meningitis. Therefore, reliance on ancillary tests such as CSF Cryptococcal Antigen/PCR and culture was crucial for diagnosis. Furthermore, our center is a resource-limited secondary district hospital, and we do not have an MRI machine at our disposal, posing a significant challenge in managing this case.

Elevated ICP is a hallmark of CM due to CSF outflow obstruction by fungal polysaccharides (2). Phase-contrast cine MRI (PC-MRI) has been applied in CM to quantify CSF flow velocity. A higher average flow was noticed in chronic CM due to CSF malabsorption and reduced intracranial compliance, resulting in diminished arterial pulsations and heightened capillary pulsations (6). Additionally, the formation of exudates within the ventricular system corresponds to the elevation of ICP in patients with meningitis (7). Phase-contrast cine MRI studies have shown that CM survivors may exhibit near-normal flow profiles when bulk outflow pathways remain patent, even in the presence of disease-related morbidity.

Possible explanations for a normal ICP in our case include early disease development and host immunity factors. A low fungal burden may not yet have caused arachnoid granulation obstruction, thereby preserving the CSF drainage pathway (8). Furthermore, immunocompetence may limit inflammation-driven ICP elevation (9), which further explains why the opening pressure in our case remained within the normal range. In addition, basal meningitides, including cryptococcosis, can produce discordant ventricular versus lumbar CSF findings, reflecting uneven distribution of fungal elements and inflammation across CSF spaces (10). Serial MRI in CM has demonstrated perivascular and perivenular fungal accumulation within the basal ganglia and brainstem (11), a pattern that can generate regional flow disturbances and focal pressure effects while sparing global ICP elevation. In our case, preserved compliance at the arachnoid villi likely masked localized disease dynamics, underscoring the limitations of relying solely on lumbar puncture pressure to guide diagnosis and management.

Immunocompetent CM is considered a rare entity. An epidemiology study shows that only 5%–10% of CM cases occur in immunocompetent hosts (1). Therefore, one must further explore factors that may contribute to disease development in these patients, such as immune dysfunction (12). Ear, nose, and throat (ENT) infections are commonly observed among patients with cerebral venous thrombosis (CVT) (13). Mastoiditis, particularly when located near the site of thrombosis, typically, the transverse or sigmoid sinus was present in 31% of cases (13), suggesting likely contiguous spread of infection. However, it remains uncertain whether mastoiditis or meningitis is the primary cause of CVT in these instances.

Additionally, our case presented with the detection of superior sagittal sinus thrombosis (SSST) on CECT brain, a relatively peculiar association with CM. This radiological finding correlated with frontoparietal cortical edema as observed in a previous study (14), consistent with venous congestion in the drainage territory of the SSST. Basal meningeal inflammation can extend to dural venous structures, and *Cryptococcus neoformans* has been shown to adhere to endothelial cells, trigger pro-inflammatory cytokines, and promote a hypercoagulable state. The dual insult: local infection and venous outflow obstruction likely explains the simultaneous parenchymal changes and supports the need for early venous imaging (CECT/MRI) in cryptococcal meningitis.

A case report revealed a positive test for hyperhomocysteinemia and methylenetetrahydrofolate reductase (MTHFR) variants (C677T and A1298C) in a patient diagnosed with meningococcal meningitis (15). In one report, anticardiolipin antibody was the most common prothrombotic risk factor (16), while in another, the homozygous MTHFR mutation was the most common (17). A German study reported cases in which either elevated lipoprotein A or protein C deficiency was associated with CVT (18). In an American study, protein C and antithrombin III deficiencies were common (19). Other potential procoagulant markers recently considered in CVT include anemia, hyperhomocysteinemia, and factor VIII levels (20). Further evaluation of a patient’s prothrombotic status should be done in cases with an obvious cause. However, the patient’s family refused further investigations due to the additional costs involved, citing financial constraints on their part.

Our mechanistic hypothesis that CM and CVT co-occur through localized endothelial invasion, cytokine-mediated hypercoagulability, and virulence factor–driven endothelial dysfunction is supported by several lines of evidence. *In vivo* models demonstrate *Cryptococcus neoformans* trapping within cerebral vessels, proliferation, and vessel rupture—providing a direct route to vascular damage (21). Simultaneously, CSF profiles in CM consistently show elevated IL-6, IL-17, and other cytokines that can induce endothelial activation and prothrombotic states (22). Finally, cryptococcal ability to activate endothelial signaling underscores a capacity to disrupt endothelial function and homeostasis (23).

The choice of amphotericin and fluconazole reflects current world health organization (WHO) and infectious diseases society of America (IDSA) guidelines for CM management in resource-limited settings when flucytosine is unavailable. Despite the absence of flucytosine, an agent associated with superior outcomes, the selected regimen can elucidate positive results in this context. Given the confirmed CVT, anticoagulation with low molecular weight heparin (LMWH) was initiated at a therapeutic dose and was later transitioned to oral anticoagulation once the patient was clinically stable for discharge.

Although anticoagulation and supportive care are the main treatments, the timely administration of anticoagulants remains challenging due to diagnostic dilemmas. Early recognition of the condition is crucial to avoid further complications. Sepsis-related thrombosis in the superior sagittal and cavernous sinuses is particularly linked to a markedly high mortality rate (13). A case from China described an immunocompetent pregnant woman diagnosed with CM complicated by CVT (24). Despite aggressive anticoagulant and antifungal therapy, the patient progressed to septic shock, multiple organ failure, and ultimately brain herniation. This highlights the need for a high index of vigilance for potential CM, despite a normal OP. Finally, early use of MR venography (MRV) enabled prompt diagnosis, while susceptibility-weighted imaging (SWI) provided complementary visualization of cortical vein thrombi, supporting recent recommendations for early venous imaging in CM with refractory headache or focal deficits.

## 4 Conclusion

This case highlights that CM can present with normal opening pressure (OP) even in immunocompetent patients, challenging the dogma that elevated ICP is universal. It underscores that CM should not be excluded based on OP alone. Furthermore, management of increased ICP should be individualized based on the patient’s symptoms, as a common headache may reflect a non-ICP-related pathology (e.g., CVT). Clinicians should also investigate host factors such as environmental exposure, subtle immunodeficiency, or prothrombotic state that may explain atypical presentations. Regardless, the classic causality dilemma, whether CVT leads to CM or vice versa, is still uncertain. Overall, the association between thrombosis and cryptococcal meningoencephalitis in immunocompetent individuals is not clearly understood, and it remains unclear whether thrombosis is a cause or a consequence. We hope this manuscript will pave the way for further studies on this topic.

## Patient perspective

“I had been having headaches for days and thought it was just stress. When they told me I had a fungal infection in my brain and a clot, I was terrified. The treatment was tough, but I’m grateful the doctors figured it out early. Now I’m back to my normal life and taking my medicines as advised.”

## Data Availability

The original contributions presented in the study are included in the article/supplementary material, further inquiries can be directed to the corresponding author.

## Appendix: care-compliant timeline

**Table:**

Date (2023)	Event/symptoms	Investigations and key findings	Management/interventions
26 September–2 October (Day −7 to −1)	Fever (3 days), right-sided throbbing headache (7/10), blurred vision, photophobia, neck pain	No medical evaluation sought; self-medicated with paracetamol	None
3 October (Day 0 – Presentation)	Presented to ED: BP 150/91, HR 95, afebrile, neurologically intact	Blood: Hb 17.2, TWBC 10.8, Plt 192, CRP 9.58, renal and liver function normal, viral serology (HIV, Hep B/C) negative; ANA, APL negative	Admitted, started on IV Rocephin 2 g BD, IV Acyclovir 500 mg TDS, S/C Clexane 60 mg BD
3 October (Evening)	Persistent headache	CECT Brain: *Superior sagittal sinus thrombosis*	Continued LMWH
4 October (Day +1)	Clinical suspicion of meningitis	*Lumbar puncture:* Opening pressure 15 mmHg; CSF sent for analysis	Awaited results
6 October (Day +3)	CSF Cryptococcal PCR *positive*	CSF: Cryptococcal antigen detected, fungal PCR positive	*Started IV Amphotericin B* + *Oral Flucytosine*
8 October (Day +5)	Persistent headache	Repeat LP by another operator: *OP 8 mmHg*	Continued antifungal therapy
20 October (Day +17)	End of induction phase	CSF culture cleared; patient clinically improved	Discharged with *Fluconazole 800 mg OD (8 weeks)* → then *200 mg OD (1 year)*; continued anticoagulation
Follow-up (November 2023 – ongoing)	No recurrence of headache or neurological symptoms	Outpatient review: stable	Maintenance antifungal therapy continues
